# Synthesis of large single-transcript pathways from oligonucleotide pools: Design of STARBURST, an autobioluminescent reporter

**DOI:** 10.1073/pnas.2508109122

**Published:** 2025-07-29

**Authors:** Gony Dvir, Zenan Xing, Irina Beldman, Andrés Rivera, Ian Wheeldon, Sean R. Cutler

**Affiliations:** ^a^Center for Plant Cell Biology, University of California Riverside, Riverside, CA 92521; ^b^Institute for Integrative Genome Biology, University of California Riverside, Riverside, CA 92521; ^c^Botany and Plant Science, University of California Riverside, Riverside, CA 92521; ^d^Centro de Ciencias Genómicas, Universidad Nacional Autónoma de México, Cuernavaca 62210, Mexico; ^e^Chemical and Environmental Engineering, University of California Riverside, Riverside, CA 92521; ^f^Center for Industrial Biotechnology, University of California Riverside, Riverside, CA 92521

**Keywords:** bioluminescense, gene synthesis, synthetic biology, plant biology

## Abstract

Methods for fast and inexpensive gene synthesis from oligonucleotide pools enable rapid iteration of genetic designs. Here, we describe *iggypop* (indexed Golden Gate gene assembly from PCR-amplified oligonucleotide pools), a simple computational–experimental pipeline that allows for low-cost design and synthesis of hundreds of genes from oligonucleotide pools using Golden Gate assembly methods. We used *iggypop* to synthesize a series of single-transcript autonomously bioluminescent reporters (STARBURSTs) that link the five genes of a fungal bioluminescence pathway via ribosomal skipping LP4/2A sequences into a 9.5 kb transcript that function in planta. We also synthesized RUBY reporters (a reporter gene system producing red betalain pigment) recoded to match dicot codon usage, as RUBY was codon optimized for rice codon usage and has a high GC content. Surprisingly, the recoded RUBYs substantially reduced betalain production in transient **Nicotiana* benthamiana* assays. Based on this observation, we synthesized six GC-boosted STARBURSTs, which produced robust luminescence in both transient assays and transgenic *Arabidopsis* plants. Thus, *iggypop* enabled the rapid synthesis of multiple genetic designs to deliver a bright single transcript autobioluminescent reporter. *Iggypop* should enable the facile synthesis and optimization of new genetic parts and complex polycistronic pathways.

## Significance

Large-scale DNA synthesis from oligonucleotide pools is valuable for synthetic biology as it enables low-cost gene synthesis. Here, we developed a simple computational–experimental pipeline, *iggypop*, that enables rapid gene synthesis and exploration of design space, a critical need in plant synthetic biology. We used it to build a series of single-transcript autobioluminescent reporters (STARBURSTs) that link the five enzymes of a fungal bioluminescence pathway in a single transcript using LP4/2A linkers. The low cost and ease of *iggypop* allowed us to rapidly test several genetic designs to yield a bright reporter for facile measurements of plant gene expression and illuminated an unappreciated role of GC content in plant synthetic gene design. Thus, *iggypop* accelerates the development of new genetic parts.

Gene synthesis is fundamental to synthetic biology and underpins the creation of modular genetic parts (1). Although gene synthesis costs have decreased substantially and multiple prokaryotic genomes have been synthesized, costs remain orders of magnitude higher per base than the per-base cost of oligonucleotides synthesized in pools. This gap makes custom synthesis of large numbers of genetic parts costly, which in turn can limit the number of designs tested. Multiple design iterations are often required to make well-functioning genetic parts, and this is particularly true for plant synthetic biology, where the rules for effective gene design are less clear than for microbial systems. Low-cost gene synthesis methods can enable greater design space exploration and accelerate the discovery of important design factors, which has spawned diverse efforts to leverage the low cost of oligonucleotide pools for genetic parts design (2–5).

Plant synthetic biology faces different constraints than many other systems. Transfer DNA (T-DNA) cassettes delivered by disarmed Agrobacterium strains are the primary means of introducing genetic constructs into plants. The largest T-DNA sizes delivered to plant cells are approximately ~150 kb (6); in practice, T-DNA constructs greater than ~30 kb are uncommonly built due to the relatively low efficiency of recovering complete insertion events. In addition, a relatively small number of well-validated promoters and terminators are available as standardized parts, which can make multigene constructs that lack repetitive sequences challenging to create and have spawned recent efforts to design and mine new regulatory elements (7, 8). Ribosomal skipping peptides, used widely across biological systems, have been adopted in plant synthetic biology to link multiple coding sequences into single polycistronic transcription units (9, 10). Up to four genes have been successfully linked by 2A peptides in plants (11, 12). There are suggestions that transcript size can limit the success of this approach in plants (11). However, up to nine genes have been successfully linked in a 12 kb coding sequence in *K. phaffii* (Pichia) (13). Polycistronic messenger RNAs (mRNAs) allow the design of compact pathways that use fewer regulatory elements relative to monocistronic design and are useful for addressing some of the challenges of expressing multigene pathways in plants.

To facilitate iterative genetic design, we developed *iggypop* (indexed Golden Gate gene assembly from PCR-amplified oligonucleotide pools), a simple computational–experimental pipeline for building complex genes and sequences. We also employed the high-throughput and cost-effective Nanopore sequencing technology to validate the accuracy of the assemblies. *Iggypop* leverages oligonucleotide pools, Golden Gate assembly, and Nanopore amplicon sequencing to streamline the creation of multikilobase DNA fragments. We used *iggypop* to develop STARBURST, a single-transcript autobioluminescent reporter created by linking the five genes of a fungal bioluminescence pathway with LP4/2A ribosomal skipping peptides in a 9.5 kb coding sequence. *Iggypop* allowed us to synthesize and test multiple designs and uncover the impact of GC content on the expression of synthetic plant genetic parts.

## Results

### IGGYPOP Enables Rapid and Accurate DNA Assembly.

**Fig. 1.**
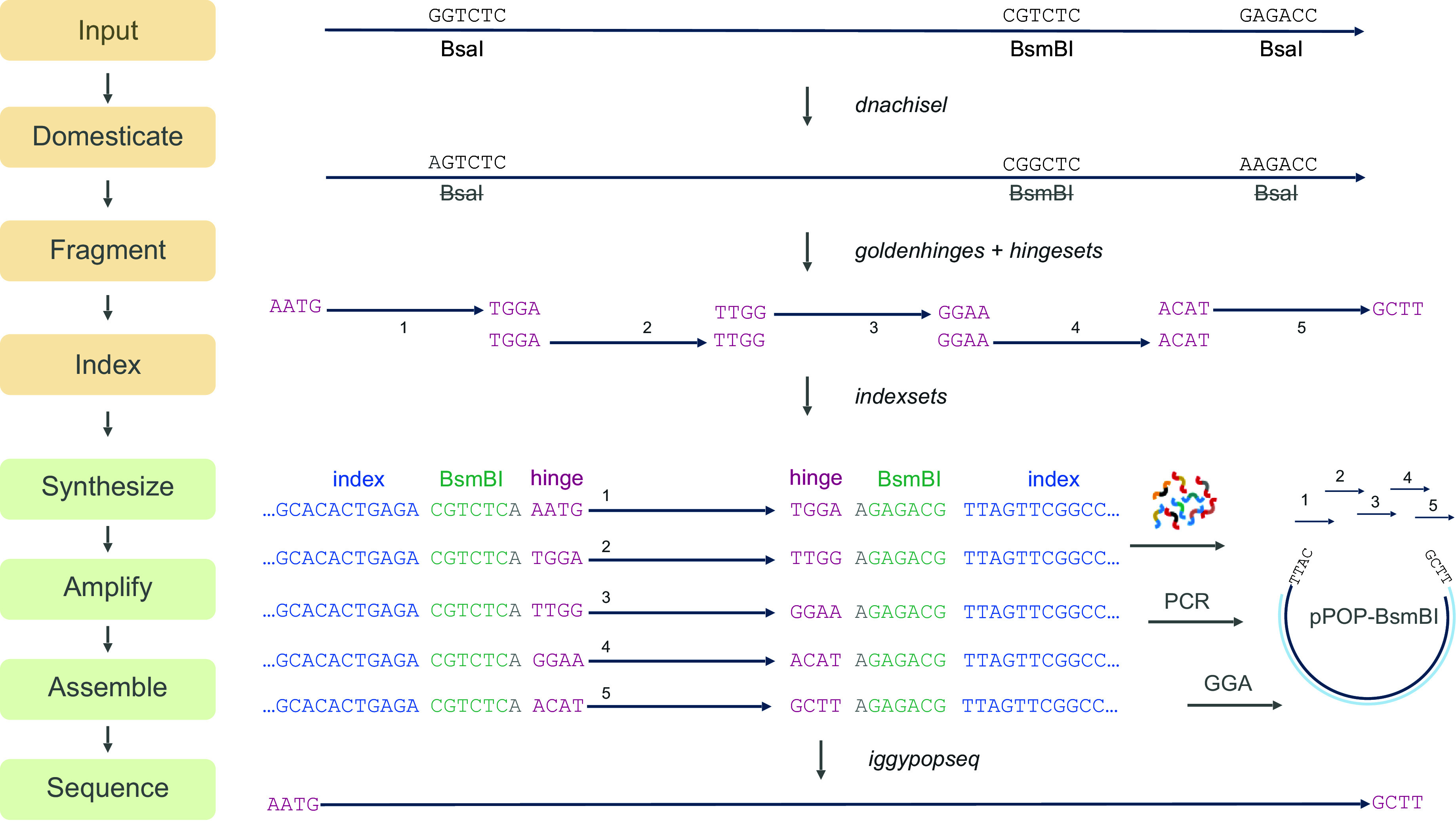
Gene assembly from oligo pools with *iggypop*. The gene assembly pipeline begins with a set of target sequences, which are domesticated using the software package *dnachisel* to remove restriction sites. The sequences are then broken into fragments with overhang “hinges” for reassembly using Golden Gate cloning. High-fidelity overhangs are selected from a large collection of precomputed high-fidelity *hingesets*. Once hinged, the fragments are indexed with index and restriction sequences to enable parallelized amplification of all fragments required for a gene’s assembly. For the work described here, we synthesized the fragments as ~250 bp oligonucleotides; 50 bp of each is used for index and cut sequences. Once assembled and cloned, colonies are sequenced using a nanopore amplicon sequencing pipeline to identify the final sequence-verified clones.

To empower large-scale gene synthesis projects and accelerate construct creation by direct synthesis, we created an open-source software tool and experimental gene-synthesis pipeline—*iggypop*—which enables rapid gene assembly from oligonucleotide pool templates (Fig. 1). Our approach is similar to recently described methods for gene synthesis from oligonucleotide pools (3, 4). The pipeline begins by computationally cutting input sequences into small fragments at sites with efficient 4-base overhangs for reassembly by Golden Gate cloning; experimentally determined all-by-all overhang ligation data (14) is used to guide the overhang selection. The fragmented sequences are then appended with type IIS restriction enzyme cut sequences and unique primer-binding sequences (indexes) that allow all fragments for a target to be amplified from oligonucleotide pools in a single-tube reaction. Error-free clones are identified after Golden Gate assembly by sequencing barcoded amplicons. The code for this pipeline is available at https://github.com/cutlersr/iggypop.

**Fig. 2.**
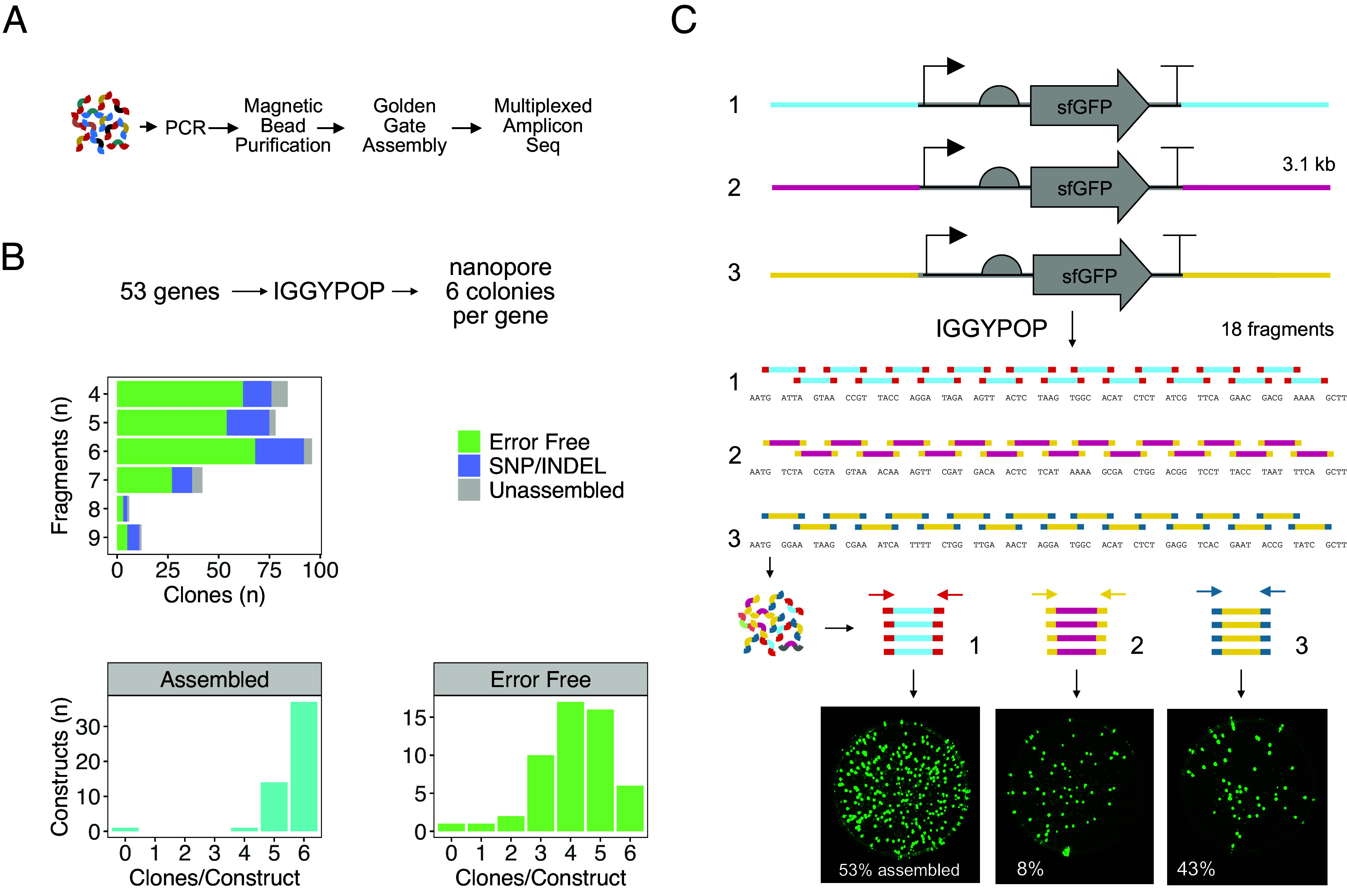
Validation of the IGGYPOP assembly method. (*A*) Iggypop experimental workflow. After computational design, oligo fragments are amplified by PCR from oligonucleotide pools, purified on magnetic beads, and assembled. After transformation, individual colonies are PCR-amplified and amplicon sequenced. (*B*) Analysis of 53 transcription factor genes assembled using IGGYPOP. The genes synthesized were assembled from between 4 and 9 fragments; amplicons from six colonies per gene were amplified using barcoded primers and sequenced using Oxford Nanopore Technologies, and the data were processed using the *iggypopseq* pipeline. The upper histogram shows the distribution of the sequencing outcomes; 93% of amplicons contained correctly assembled fragments, and 69% contained error-free sequences. The *Bottom* two panels show the outcomes per target gene synthesized; correctly assembled and error-free sequences were obtained for at least one amplicon for 52 of the 53 targets synthesized. (*C*) Schematic representation of IGGYPOP assembly for three ~3 kb constructs, each containing a GFP expression cassette. The sequence of the central GFP cassette (shown in gray) is fixed, and the outer flanking ~2 kb of each clone is distinct between the three constructs to create different assembly overhangs (shown underneath each construct’s set of 18 assembly fragments). The designed constructs were fragmented into indexed assembly blocks using *iggypop*, synthesized in oligonucleotide pools, amplified using construct-specific index primers, assembled by Golden Gate, and transformed into *E. coli*. Representative colony plates are shown at the *Bottom*; these indicate 53%, 8%, and 43% assembly efficiencies for the three constructs.

To test the pipeline, we designed and synthesized 53 phylogenetically diverse plant transcription factors using a simple workflow (Fig. 2 *A* and *B*). We used *iggypop* to design a collection of domesticated, fragmented sequences that could be reassembled from a pool of 292 indexed oligonucleotides. The set of targets had a median size of 923 bp, and its members were assembled by 4 to 9 oligos. The oligonucleotides were PCR-amplified with gene-specific index primers, reaction products were purified using magnetic beads, and the assembly was performed using Golden Gate reactions, followed by transformation into *Escherichia coli*. Six amplicons or clones per construct were amplified from colonies using barcoded vector primers, pooled, and nanopore sequenced (see *Methods* for details and the analysis pipeline). In this experiment, 93% of the constructs were correctly assembled, 69% of the clones sequenced were error-free, and at least one error-free clone was obtained for 52 of the 53 targets (Fig. 2*B*). To establish whether we could routinely assemble larger targets built from more fragments, we designed a series of three ~3,100 bp sequences harboring a central Green Fluorescent Protein (GFP) expression cassette, each of which was assembled from 18 oligonucleotides with different overhang sets; these displayed 53%, 8%, and 43% assembly efficiencies (Fig. 2*C*). It is unclear why design 2 performed worse than designs 1 and 3; the designs have predicted ligation fidelities between 97.3% and 97.8%. Nonetheless, these data demonstrate the assembly of up to 18 fragments, albeit with sometimes marginal efficiency that would necessitate substantial amplicon sequencing to identify error-free clones.

### Two-Step Assembly of STARBURST via IGGYPOP.

**Fig. 3.**
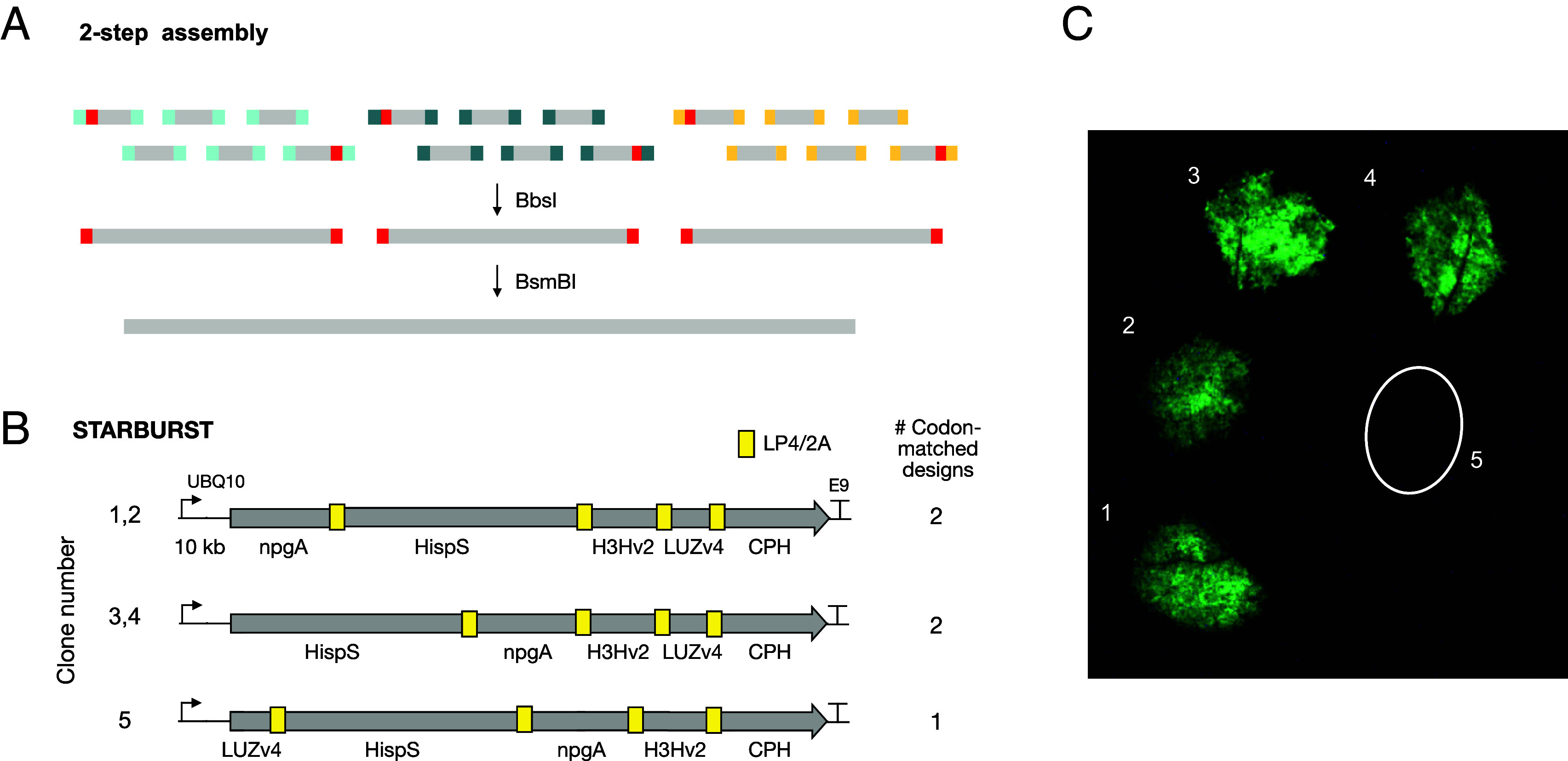
Construction of single transcript bioluminescence reporters. (*A*) Two-step *iggypop* gene assembly strategy. A target sequence (*Bottom*) is divided into small segments at junctions predicted to assemble with high fidelity; the segments are flanked with BsmBI sites, which are used for the second step assembly. The fragments are then synthesized separately from oligonucleotide pools, with each oligo flanked by BbsI sites, which are used for the first-step assembly reactions. (*B*) Design of single-transcript autonomously bioluminescent reporters. The figure shows the variants designed and synthesized using three different gene orders. The constructs link the five genes of the optimized FBP3 pathway with LP4/2A ribosomal skipping peptides. Two different *Arabidopsis* codon-matched variants were created for the first two designs, and a single codon-optimized sequence was used for the bottom design. (*C*) Transient expression of clones 1 to 5 in *N. benthamiana* leaves. Luminescence was observed in four of the five constructs; the images shown were acquired 4 d after coinfiltration with p19. The image was acquired using a Sony A7S (ILCE-7S) with an FE 90 mm F2.8 Macro G OSS lens at f2.8 aperture, 30 s exposure, and 6400 ISO.

Having established the pipeline, we used it to design plant reporter constructs, focusing on a fungal bioluminescence pathway (15–18) that enables autonomous gene expression reporting. Multiple improvements have been made since the discovery of the pathway (17), but the complexity and size of the five transcription units make it challenging to work with. Inspired by the success of the RUBY reporter (19), which links the three-gene betanin pathway into a single transcript, we tested whether we could build a single-transcript autonomous bioluminescence reporter system (STARBURST) by linking the pathway’s five enzymes with intervening LP4/2A ribosomal skipping peptides (20–22). We designed and synthesized constructs with three different gene orders in a two-step process (Fig. 3*A*). The ~9.5 kb coding sequences were computationally segmented into nine ~1,050 bp segments at junctions with high-fidelity assembly overhangs; these “step-one” blocks were then synthesized separately from oligonucleotide pools, sequence validated, and assembled in a second step to yield the final targets. Using this approach, we created multiple codon-optimized versions of designs that place the longest gene (*HispS*; ~5 kb) in the first or second position (Fig. 3*B*). Codon usage was optimized to match *Arabidopsis* usage. Four of the five polycistronic genes produced luminescence in transient assays (Fig. 3*C*), demonstrating that single-transcript versions of the pathway are functional.

### Higher GC Content Boosts STARBURST Expression.

**Fig. 4.**
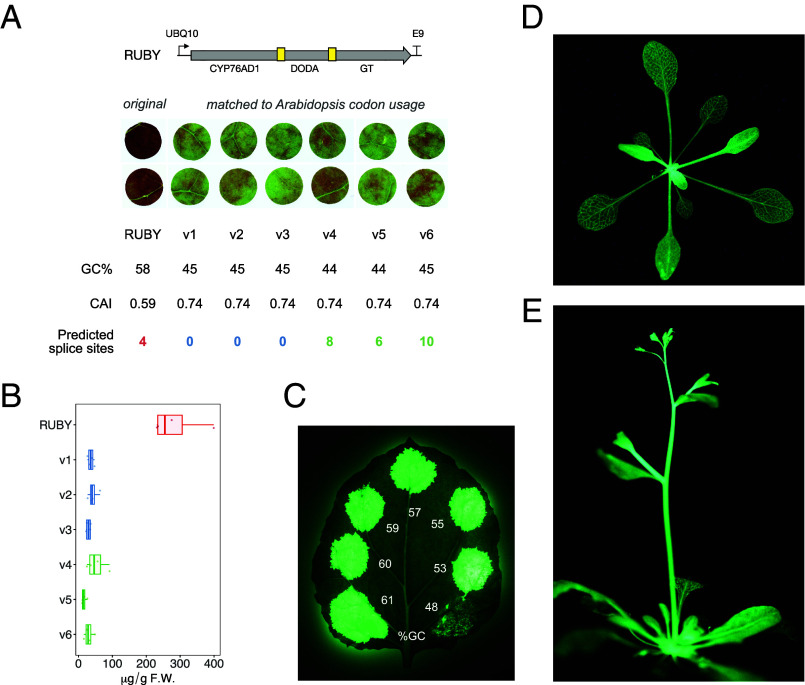
Effect of GC content and predicted splice junction elimination on synthetic gene expression. (*A* and *B*) Low-GC-content RUBY variants have reduced pigment production in *N. benthamiana* transient assays. Transient expression of RUBY and six *Arabidopsis*-codon-matched variants in *N. benthamiana.* RUBY (GC content: 56%) produced robust pigmentation, whereas the codon-matched variants (GC content ~45%) showed substantially reduced pigmentation. *Inset* are the GC content of each coding sequence, codon adaption index values (using an *Arabidopsis* codon table), and the number of splice site donors and acceptors predicted using *Spliceator* (24); predicted splice junctions were purged from three of the six designs (v1 - v3); all six designs match *Arabidopsis* codon usage. Images were acquired 2 d postinfiltration; infiltrations were conducted using p19 coinfiltrations. (*B*) Quantification of pigmentation levels for RUBY and codon-matched variants shown in panel (*A*); pigment quantifications were conducted after ethanol extraction of leaf discs 48 h postinfiltration, as described in reference (25). (*C*) GC-boosted STARBURSTs produce high-level luminescence in *N. benthamiana* transient assays. Six coding sequences for design # 2 (HispS-npgA-H3H-Luz-CPH; Fig. 3) were designed using *iggypop* with varying GC contents and cloned into pPLANT-POP to create UBQ10-driven constructs; all designs were also “deintronized.” The figure shows images collected 3 d post–coinfiltration of the clones with p19. (*D* and *E*) Transgenic T1 *Arabidopsis* plant expressing UBQ10::STARBURST (53% GC content), Line #15 (*D*) and Line #9 (*E*). Images *C*–*E* were acquired using a Sony A7S (ILCE-7S) with an FE 90 mm F2.8 Macro G OSS lens at f2.8 aperture, 30 s exposure, and 6400 ISO and are unmodified JPEGs.

The expression of coding sequences moved between organisms can be low due to inappropriately spliced cryptic introns, as illustrated by the original attempts to express *A. victorialis* GFP in plants (23). Synthetic coding sequences, particularly those that are long, may benefit from removing potential intronic sequences, which can be predicted using machine learning models (24). We incorporated a “deintronization” function into *iggypop* to identify and replace predicted intron acceptor and donor sites with synonymous mutations. We used the 4.2 kb RUBY reporter gene as a test case for this approach and synthesized six *Arabidopsis*-codon-matched sequences, three of which were purged of predicted junctions, along with a control RUBY sequence. These were tested in transient *N. benthamiana* assays (Fig. 4*A*). These experiments do not suggest a benefit of eliminating predicted splice junctions, at least for our small set of RUBY test sequences; however, we surprisingly observed that all of the designed sequences produced ~5 to 10-fold less pigment than the RUBY control, suggesting that our design process, which had matched *Arabidopsis* codon usage for all of the test genes, had reduced their effectiveness (Fig. 4*B*).

Inspection of RUBY’s sequence indicated that its GC content is much higher than our designed sequences—58% GC content for RUBY and 44 to 45% for our designed sequences (Fig. 4*A*). RUBY’s three enzymes originated from the dicotyledonous plant *Beta vulgaris* and possess similar GC and GC_3S_ content to *Arabidopsis* genes (*SI Appendix*, Fig. S2). However, RUBY’s sequence was designed to match rice GC-rich codon usage (19), which yields a low codon adaptation index, an indicator of preferred codon usage, for *N. benthamiana* despite its highly efficient expression in eudicots (*SI Appendix*, Fig. S1). Studies across multiple biological kingdoms have reported that elevated GC content in CDSs can enhance the expression of engineered constructs (26). The mechanistic basis for this is unclear, but it has been associated with increases in steady-state transcript levels, mRNA export to the cytoplasm, reductions in premature transcript termination, improved mRNA translation, reduced localization to P-bodies, and reduced silencing (26–32).

We, therefore, designed and synthesized six new constructs with GC contents ranging from 53 to 63% and compared these to one of our *Arabidopsis*-codon-matched sequences with a 48% GC content. The GC-boosted clones showed increased luminescence in transient *N. benthamiana* assays (Fig. 4*C*). Although the underlying basis of this improvement is unknown, this experiment demonstrates that synthetic plant gene design can benefit from testing designs with a range of GC contents and varying codon usage. We selected the construct with 53% GC content, which we refer to as STARBURST, for validation in planta. Transgenic *Arabidopsis* T1 plants harboring a UBQ10::STARBURST construct exhibit bright luminescence in leaves, stems, and flowers (Fig. 4 *D* and *E*).

## Discussion

We have developed iggypop, a simple and efficient pipeline for designing and synthesizing genes from oligonucleotide pools using Golden Gate cloning methods and Nanopore amplicon sequencing. Using this pipeline, we synthesized ~200 kb of DNA at materials costs of ~$10 per 1,000 bp (Dataset S1), with an additional ~$20 per two-step clone, enabling us to iterate on gene designs to improve reporter gene expression rapidly and observed that high GC content is important for RUBY’s high betalain production and that this can be used to enhance synthetic gene expression. This work complements recent methods for gene assembly and barcoding from oligonucleotide pools (3, 4, 33). It provides a simple, end-to-end pipeline for rapid gene synthesis, facilitating the construction of genetic parts.

Inspired by the success of the RUBY reporter, which links the three-gene betalain pathway into a single transcript, we used *iggypopp* to develop STARBURST. This optimized single-transcript bioluminescence reporter links the five genes of the fungal bioluminescence pathway via LP4/2A ribosomal skipping peptides into a 9.5-kb transcript. Unlike multitranscript systems, STARBURST simplifies the use of autonomous bioluminescence by requiring only one promoter and terminator. It functions in transient *N. benthamiana* assays and stable *Arabidopsis* lines, making it broadly useful for tracking gene expression in plant systems. To our knowledge, STARBURST contains the most genes linked by 2A peptides that have been successfully expressed in planta. Prior work has linked up to four carotenoid biosynthetic genes into bi-, tri-, and quad-cistronic transcripts; while the bicistronic and tricistronic constructs generally produced the expected proteins, the 4.1 kb quad-cistronic construct showed incomplete “cleavage” of its last two encoded proteins and reduced carotenoid production (11). In *K. phaffii*, up to nine genes have been successfully linked in a 12 kb coding sequence (13). Our results suggest that longer and more complex polycistronic transcripts may be possible in plants and that such design efforts can benefit from testing different gene orders and GC contents. STARBURST demonstrates that large, polycistronic constructs can be made functional in plants, opening the door to more compact metabolic engineering strategies. The ability to rapidly iterate through many designs at a relatively low cost should accelerate the design of complex polycistronic metabolic pathways in plants and help elucidate their design rules.

## Methods

### Sequence Domestication and Codon Optimization.

*iggypop* includes pipelines for processing fasta and Genbank formatted sequences into oligonucleotide pools; the fragments for each target gene are amplified with a unique primer pair and assembled using Golden Gate methods. It uses *dnachisel* (34), a Python library of sequence optimization functions, to optimize and domesticate sequences. Codon optimization uses either Kazusa or the more current CoCoPUTs dataset of codon tables (35). All sequences were designed using *UniquifyAllKmers* optimization parameter to prevent sequence repeats of 12 or greater nucleotides, *AvoidHairpins* constraint to preclude hairpins of 20 nucleotides or greater over a 250 bp window, and *AvoidPattern* constraints to preclude common IIS restriction site sequences. *Iggypop* uses YAML files to specify *dnachisel* parameters used for sequence optimizations. *iggypop* installation and usage information is provided in the *iggypop* GitHub repository (https://github.com/cutlersr/iggypop).

### High-Fidelity Overhang Sets.

High-fidelity overhang sets are used to improve multifragment Golden Gate ligations. We achieved this by selecting hinge overhangs from precomputed high-fidelity sets (*hingesets*), which are used to constrain overhang selections by *goldenhinges*—a Python package that fragments sequences at junctions that produce unique hinge sets within a specified radius of targeted cut sites (36). The predicted ligation fidelities of hinging solutions generated using this approach are as good or better than the fidelity of the constraining set from which the solution is selected. We created our *hingesets* using a genetic algorithm optimizer to create high-fidelity sets of varying sizes, scoring fidelities using Potapov et al. (14) data; all sets included 5′-AATG-3′ and 5′-GCTT-3′, which are used for cloning into pPOP vectors. We note that AATG & GCTT have a near-perfect predicted ligation fidelity and are compatible with GoldenBraid 2.0 and MoClo coding sequence rules (37). When tested against 4 K plant cDNAs, *hingesets* produced a predicted median fidelity of 99.8% (*SI Appendix*, Fig. S2).

### Indexing Primers.

To create indexing primers, we randomly generated 50% GC, restriction site-free sequences as *primer3-py* (38) templates, with their parameters set to generate 18-mers with 60 °C T_m_. We filtered these to create a final set of primers with reduced potential to cross-hybridize to other index primers and reduced potential to amplify common lab contaminant DNAs using *MFEprimer-3* (39) to remove primers predicted to form hairpins or primer dimers. The remaining ~700 K primers were then scored for pairwise cross-hybridization using *MFEPrimer3*, and the 10 K primers with the lowest cross-hybridization scores (*indexset*) were retained (Dataset S1).

### pPOP Vectors.

The pPOP vectors produce 5′-AATG-3′- and 5′-GCTT-3′-compatible overhangs when digested with either BsmBI or BbsI; these were created by modifying pUPD2 (40) to add cloning sites and an intervening AmilCP *E. coli* expression cassette to enable blue/white insert screening [derived from mUAV (41)]. These vectors were built using PCR and Golden Gate cloning. pPlantPOP was designed similarly to include an AmilCP marker using the GoldenBraid collection of parts (40); it is in a pCAMBIA backbone and allows for one-step assembly from oligonucleotide pools and subsequent functional analyses *in planta*. pPlantPOP contains an AtUbq10p:BsmBI-AmilCP-BsmBI:E9-terminator cloning cassette and NOSp:NPTII & 35Sp:DsRed selection/reporter cassettes. The pPOP vectors were confirmed by whole-plasmid sequencing (Azenta Life Sciences, USA; *SI Appendix*, Table S1; https://bit.ly/45LMTLg, https://bit.ly/44V0cqK, https://bit.ly/3FO9Gvq).

### Gene Assembly.

Oligonucleotide pool templates were synthesized by Twist Bioscience (USA), resuspended to 1 ng/µL in sterile ddH_2_O, and used in PCRs using Phusion High-Fidelity DNA Polymerase (New England Biolabs, USA) in 25 µL reactions with 4 pg template, 1 µM each index primer, 200 µM deoxynucleotide triphosphates, and 1× high-fidelity (HF) buffer. PCR was performed using an initial denaturation at 98 °C (30 s), followed by 30 cycles of 98 °C (10 s), 60 °C (30 s), and 72 °C (30 s). The PCR products were purified using Sera-Mag SpeedBeads (Cytiva) or MagMAX Pure Bind Beads (ThermoFisher Scientific, USA). MagMAX-Bead purifications were conducted according to the manufacturer’s protocol, using 40 µL of bead suspension per PCR; the purified fragments were eluted in 15 µL sterile ddH_2_O and quantified using a Nanodrop Spectrophotometer (ThermoFisher, USA), with recoveries typically yielding 10 to 40 ng/µL. Sera-Mag SpeedBead purifications were conducted according to a literature protocol (42), using 50 µL of bead suspension per 25 µL PCR; the purified fragments were eluted in 25 µL sterile 10 mM Tris-HCl (pH 8.0) prewarmed to 50 °C with recoveries of ~10 to 30 ng/µL.

The purified fragments were assembled in reactions using 35 ng pPOP-BsmBI or 60 ng pPlantPOP, using ~5.5 ng per fragment (i.e., 33 ng of a purified pool with six fragments) in 10 µL reactions using a NEBridge BsmBI-v2 master mix (New England Biolabs, USA) according to the manufacturer’s protocol. The reactions were run in a thermal cycler for 90 cycles at 42 °C, then 16 °C, and a final inactivation at 60 °C for 5 min. Transformations were conducted in 96-well thin-walled PCR plates in a thermal cycler. 2 µL of each ligation was added to 50 µL chemically competent homemade Top10 *E. coli* cells, incubated on ice for 30 min, heat-shocked in the thermal cycler at 42 °C (60 s), and transferred to ice for 5 min. Each transformation was then transferred to a deep 96-well plate into 250 µL SOC and incubated with shaking (200 rpm) for 1 h (37 °C), then plated onto either 6-well or 9 cm LB + chloramphenicol (it is spectinomycin for pPlant-POP, chloramphenicol is for the pUPD versions) (25 mg/L) Petri plates.

### Amplicon Barcode Design.

We used custom barcoded primer pairs to perform colony PCR of candidates from pPOP vectors and sequenced the amplicons using Oxford Nanopore Technology (Oxford, UK). To enable this, we designed a set of 96 18-mer primer barcodes with a minimal edit distance of 8 (average ~10); we appended these to forward and reverse primer pairs flanking pPOP cloning sites, synthesized them (IDT, USA), and used them in colony PCRs. The barcodes were generated using *iggypop.py primers* to create a set of candidate 18-mer primers, which were processed to identify a maximally different subset using *barcode_selection.py,* available in the *iggypop* repository. The barcodes for the pPOP and pPlantPOP vectors are provided in Dataset S1.

### Nanopore Amplicon Sequencing.

We PCR-amplified assemblies from colonies using GoTaq DNA Polymerase (Promega, USA) in 10 µL reactions with 0.1 µM each barcoded primer. 2 µL of each reaction was combined into a pool, bead-purified using an equal volume of MagMAX or 2× volume of Ser-Mag beads. The barcoded amplicons were resuspended to a final concentration of ~100 to 500 ng/μL. A total of 1.5 µg of the pooled products were then end- repaired using NEBNext (New England Biolabs, USA), purified using an equal volume of MagMAX and ligated to adapters using the Ligation Sequencing Kit XL V14 (Oxford Nanopore Technologies, USA). The final libraries were quantified using a Qubit. 50 fmol (~60 ng) was then analyzed using a MinION Flow Cell R10.4.1 (Oxford Nanopore Technologies, USA). The data obtained were processed using *iggypopseq*, a pipeline we developed based on previous research (43–45). Briefly, it uses *minibar* (46) to demultiplex reads, *chopper* (47) to remove low-quality reads, *minimap2* (48) to map reads to reference sequences, and *samtools* (49), *bcftools* (49), *bedtools* (50), *racon* (51), *medaka* (52), *seqtk* (53), *emboss* (54), and *parallel* (55) to generate consensus sequences, annotate variants, and output summaries. A detailed procedure is available at https://github.com/ZenanXing/Construct-Validation-for-IGGYPOPseq.

### Superfolder GFP (sfGFP) Control Assemblies.

An ~1,000-bp expression cassette composed of sfGFP, the strong J23119 promoter, B0034 ribosome binding site, and *rrnB* T1/lambda t0 terminators, was designed in Geneious; this central sequence was fixed, and three derivatives designed, flanked on each side by ~1 kb of randomly generated, 50% GC, restriction-siteless sequence. The three sequences were fragmented using *iggypop* into 18 oligonucleotides. Each of the three fragment sets uses different overhang/hinge sets due to the nonidentical arms flanking the sfGFP cassette. The oligonucleotide fragments were synthesized by Twist (USA) and assembled as described above. Assembly efficiencies were established by counting the number of GFP^+^ colonies obtained relative to the total number of amilCP^–^ colonies. Six clones per assembly were analyzed by PCR to confirm the correct insert sizes.

### Synthesis of 53 Plant Transcription Factors.

A set of 292 indexed oligonucleotides for assembling 53 phylogenetically diverse plant transcription factors was designed with *iggypop*, synthesized by Twist Biosciences (USA), and assembled into pPlantPOP as described above. Six barcoded amplicons per gene were sequenced using the *iggypopseq* pipeline (https://github.com/ZenanXing/Construct-Validation-for-IGGYPOPseq). The oligos and target sequences synthesized are provided in Dataset S1.

### Deintronization and GC Tuning.

The sequences were optimized to match *Arabidopsis* codon usage in combination with a “deintronization” protocol designed to iteratively mutate sequences of potential intron junctions predicted by using a 200 bp window and *spliceator* (24) using *dnachisel’s AvoidPattern* function to introduce synonymous mutations in up to four iterations of intron scanning and synonymous mutation. If not obtained on the first pass, acceptor/donor-free clones were selected from multiple runs. GC content was tuned using *dnachisel* functions called by *iggypop* using *EnforceGCContent* to optimize for a minimum specified GC content over a 60-base-pair sliding window with *match_codon_usage* and an *Arabidopsis* codon table. A 4× multiplier (boost) was applied to the *EnforceGCContent* optimization to ensure that GC content dominated the optimization relative to codon usage. RUBY sequences were designed to use *match_codon_usage* and an *Arabidopsis* codon table; three of the six designed sequences used the deintronization option described above. The parameter files used for these designs are provided on the *iggypop* GitHub page.

### Two-Step Assemblies.

Sequences were designed to be assembled in a two-step process. First, target sequences are modified to add BsaI sites with AATG/GCTT overhangs adjacent to the start and stop codons, yielding domesticated, GoldenBraid, and MoClo compatible level 0/level-α coding sequences (i.e., they generate 5′-AATG…GCTT-3′ hinges after BsaI digestion). These were then broken into ~1,000 base pair segments at junctions identified to produce high-fidelity overhangs and terminal BsmBI sites appended to yield step-one fragments; each fragment was then synthesized from oligonucleotides flanked by BbsI sites; PCR-amplified products were assembled using four units of BbsI-HF (New England Biolabs, USA), 200 units of T4 DNA ligase (New England Biolabs, USA) in 1× T4 DNA ligase buffer and assemblies cloned into pPOP-BbsI. After sequence validation, the step-one fragments were assembled using 0.5 nM of each of the step-one plasmids (~30 ng) in a 15 µL reaction mix with NEBridge (BsmBI-v2) enzyme mix and 90 ng of the pPlantPOP vector; the final clones were confirmed by whole plasmid sequencing (Azenta Life Sciences, USA).

### Transient and Transgenic Assays of RUBY and STARBURST Function.

We conducted transient assays in *N. benthamiana* using the AGROBEST protocol (56). *N. benthamiana* were grown in 16-h days, and leaves from 4- to 5-wk-old plants were infiltrated with Agrobacteria C58 harboring pPlantPOP-STARBURST or pPlant-POP-RUBY clones, along with strains expressing p19, with each strain present at 0.3 OD_600_ units in AGROBEST media (56). The luminescence images were acquired three or 4 d after infiltration using a Sony A7S (ILCE-7S, FE 90 mm F2.8 Macro G OSS lens) at *f2.*8 aperture, 30 s exposure, and 6400 ISO. The RUBY images shown were acquired 2 d after infiltration. Betalains were extracted from leaf discs in 50% EtOH; the absorbance of 100 µL of the solution was measured at A_538_ in a flat-bottom 96-well plate using a Tecan Spark. Pigment amounts were inferred according to reference (25). *Arabidopsis* transgenic plants were produced using Ubq10p-STARBURST (53% GC version; https://bit.ly/3FHttNc). *Arabidopsis* transformations were conducted using the floral dip method (57).

### Online Protocols.

An online protocol for our gene assembly pipeline is available at protocols.io: https://www.protocols.io/view/iggy pop-rapid-and-large-scale-dna-assembly-method-gzzpbx75p.

## Supplementary Material

Appendix 01 (PDF)

Dataset S01 (XLSX)

## Data Availability

The source code for the pipelines mentioned in the manuscript have been deposited in https://github.com/cutlersr/iggypop (58) and https://github.com/ZenanXing/Construct-Validation-for-IGGYPOPseq (59). All data are included in the article and/or supporting information.
