# Supplementary material for: Synthesis of large single-transcript pathways from oligonucleotide pools: Design of STARBURST, an autobioluminescent reporter
Source: Proc Natl Acad Sci U S A. 2025 Jul 29;122(31):e2508109122. doi: 10.1073/pnas.2508109122 (PMC12337302; doi:10.1073/pnas.2508109122)
Supplement: Supplementary file 1 — Appendix 01 (PDF) [file pnas.2508109122.sapp.pdf]

PNAS

Supporting Information for

Synthesis of large single-transcript pathways from oligonucleotide pools: design of STARBURST, an autobioluminescent reporter.

Gony Dvir, Zenan Xing[7], Irina Beldman, Andrés Rivera, Ian Wheeldon, Sean R. Cutler

Sean Cutler
Email: cutler@ucr.edu

This PDF file includes:

Figures S1 to S2
Table S1

Other supporting materials for this manuscript include the following:

Supporting data file 1

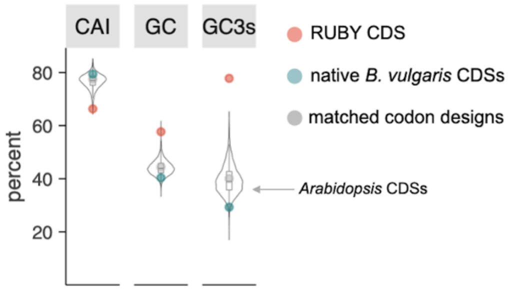

			CAI		
**Gene**	**GC**	**GC3S**	***Arabidopsis***	***Nicotiana***	***Oryza***
RUBY	57.7	77.8	0.66	0.59	0.94
B. vulgaris CDSs	40.4	29.3	0.79	0.78	0.74
mcu + deintronized 1	44.6	40.5	0.77	0.74	0.78
mcu + deintronized 2	44.6	40.5	0.77	0.74	0.78
mcu + deintronized 3	44.5	40.4	0.77	0.74	0.78
mcu-1	44.4	40.1	0.77	0.74	0.78
mcu-2	44.4	40.1	0.77	0.74	0.78
mcu-3	44.5	40.2	0.77	0.74	0.78

Fig. S1 Supplemental Figure 1. GC content and codon adaptation indices of RUBY and designed sequences. (A) Distribution of codon adaptation index values, GC content, and synonymous 3rd position GC usage ($GC3_s$) for all Arabidopsis coding sequences (violin plots) with overlays of the RUBY sequence (red dot), a concatenated sequence of the three *B. vulgaris* genes comprising RUBY (blue dot) and the designed Arabidopsis codon-matched RUBY derivatives characterized in this work (see Figure 3 in the main text). The CAI values were calculated using a genome-wide Arabidopsis codon table. (B) GC, $GC3_s$, and CAI values for RUBY and other genes. The CAI values shown were calculated using Arabidopsis, Nicotiana, and Oryza codon tables obtained from the CoCoPUTs database (1).

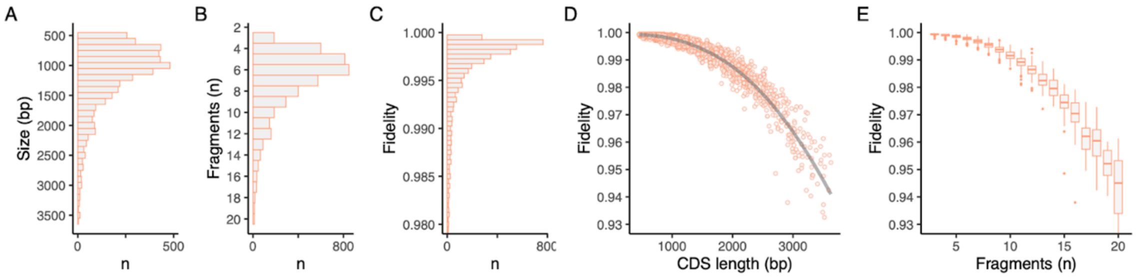

F

variable	mean	median	sd
cds_length	1184.000	1032.000	566.000
fragment_n	7.000	6.000	3.000
fidelity	0.995	0.998	0.007

Fig. S2. Prediction of multi-fragment ligation fidelities for ~4K plant coding sequence designs. A collection of ~10K transcription factor coding sequences for citrus, hemp, beet, and papaya was collated from TfDB (2). The overhang set sizes, the number of fragments required for assembly from 250 bp oligos, predicted ligation fidelities for the set, and summary statistics are shown. Predicted ligation fidelities were calculated as described in reference (3) using a data set for T4 DNA ligase and BsaI at 25 ºC for 18 hours.

Iggypop cloning vectors

pPOP-BsmBI (Addgene: 241281)
pPOP-BbsI (Addgene: 241282)
pPlantPop (Addgene: 241283)

V1 Starburst (Figure 3)

1_A01-A09_pOmega1 35Sp-DsRed-NOSt pAtUbq10-FB3_2Aified_di_mcu-1-tE9 NOS-NPTII-clone #1

1_A10-B06 pOmega1 35Sp-DsRed-NOSt pAtUbq10-FB3_2Aified_di_mcu-2-tE9 NOS-NPTII-clone #2

1_B07-C03 pOmega1 35Sp-DsRed-NOSt pAtUbq10-FBP3-2A_v2_di_mcu-2-tE9 NOS-NPTII-clone #3

1_C04-C12 pOmega1 35Sp-DsRed-NOSt pAtUbq10-FBP3-2A_v2_di_mcu-3-tE9 NOS-NPTII-clone #4

1_D01-D09 pOmega1 35Sp-DsRed-NOSt pAtUbq10-FBP3_2A_v3_di_mcu-1-tE9 NOS-NPTII-clone#5

RUBY derivatives (Figure 4)

3_D06-D09 pOmega1 35Sp-DsRed-NOSt pAtUbq10-RUBY_original_unmodified-tE9 NOS-NPTII--RUBY
3_B06-B09 pOmega1 35Sp-DsRed-NOSt pAtUbq10-RUBY_original_di_mcu-1-tE9 NOS-NPTII--v1
3_B10-C01 pOmega1 35Sp-DsRed-NOSt pAtUbq10-RUBY_original_di_mcu-2-tE9 NOS-NPTII--v2
3_C02-C05 pOmega1 35Sp-DsRed-NOSt pAtUbq10-RUBY_original_di_mcu-3-tE9 NOS-NPTII--v3
3_C06-C09 pOmega1 35Sp-DsRed-NOSt pAtUbq10-RUBY_original_mcu-1-tE9 NOS-NPTII--v4
3_C10-D01 pOmega1 35Sp-DsRed-NOSt pAtUbq10-RUBY_original_mcu-2-tE9 NOS-NPTII--v5
3_D02-D05 pOmega1 35Sp-DsRed-NOSt pAtUbq10-RUBY_original_mcu-3-tE9 NOS-NPTII--v6

GC-boosted clones -- final STARBURST clone (Figure 4)

L7_1_C01-C06 pOmega1 35Sp-DsRed-NOSt pAtUbq10-STARBURST_53gc_gc3s_50-tE9 NOS-NPTII-Final_STARBURST (Addgene: 241284)
L7_1_C07-C12 pOmega1 35Sp-DsRed-NOSt pAtUbq10-STARBURST_55gc_gc3s_55-tE9 NOS-NPTII
L7_1_D01-D06 pOmega1 35Sp-DsRed-NOSt pAtUbq10-STARBURST_57gc_gc3s_61-tE9 NOS-NPTII
L7_1_D07-D12 pOmega1 35Sp-DsRed-NOSt pAtUbq10-STARBURST_59gc_gc3s_66-tE9 NOS-NPTII
L7_1_E01-E06 pOmega1 35Sp-DsRed-NOSt pAtUbq10-STARBURST_60gc_gc3s_70-tE9 NOS-NPTII
L7_1_E07-E12 pOmega1 35Sp-DsRed-NOSt pAtUbq10-STARBURST_62gc_gc3s_75-tE9 NOS-NPTII

Table S1. Links to whole plasmid sequences of constructs used in this study.

Dataset S1. Excel file containing the sequences of the oligonucleotide pools used for gene synthesis (tabs 1 – 5), precomputed overhang sets (*hingesets*) used for selecting high fidelity overhangs (tab 6), primers used to index fragment sets for gene-specific PCR amplification (*indexsets*; tab 7), barcoding primers used for colony PCR of amplicons from pPOP and pPlant-POP vectors (tab 8), and cost estimates.

SI References

1. A. Alexaki, et al., Codon and Codon-Pair Usage Tables (CoCoPUTs): Facilitating Genetic Variation Analyses and Recombinant Gene Design. J Mol Biol **431**, 2434–2441 (2019).
2. J. Jin, *et al.*, PlantTFDB 4.0: toward a central hub for transcription factors and regulatory interactions in plants. *Nucleic Acids Res* **45**, D1040–D1045 (2017).
3. V. Potapov, *et al.*, Comprehensive Profiling of Four Base Overhang Ligation Fidelity by T4 DNA Ligase and Application to DNA Assembly. *ACS Synth Biol* **7**, 2665–2674 (2018).
